# Structural Racial Disparities in the Allocation of Disproportionate Share Hospital Payments

**DOI:** 10.1001/jamanetworkopen.2022.40328

**Published:** 2022-11-04

**Authors:** William L. Schpero, Paula Chatterjee

**Affiliations:** 1 Division of Health Policy and Economics, Department of Population Health Sciences, Joan & Sanford I. Weill Medical College, Cornell University, New York, New York; 2 Department of Medicine, Perelman School of Medicine, University of Pennsylvania, Philadelphia; 3 Leonard Davis Institute of Health Economics, University of Pennsylvania, Philadelphia

## Abstract

This cross-sectional study examines the allocation of Medicare and Medicaid Disproportionate Share Hospital payments by race.

## Introduction

Medicare and Medicaid Disproportionate Share Hospital (DSH) payment programs allocate $24 billion to hospitals annually to subsidize care and improve outcomes for low-income patients. Disproportionate Share Hospital allocations are based largely on measures of patient characteristics that reflect health care use for low-income patients, such as the proportion of inpatients enrolled in Medicaid.^1,2^

Because racial and ethnic minority groups face sizable structural barriers to health care, they often have lower levels of health care use than nonminoritized racial and ethnic groups, conditional on having the same level of health care need.^3,4,5^ Because DSH funding is partially allocated based on measures of health care use, we hypothesized that hospitals in disproportionately Black counties received payments that were incommensurate with their financial needs and the needs of the populations they served.

## Methods

For this cross-sectional study, we obtained data on Medicare and Medicaid DSH payments from the Healthcare Cost Report Information System for 2019 and State Plan Rate Year files for 2015 (the most recent years for which full data were available) for all states except Massachusetts, which does not make Medicaid DSH payments under its Section 1115 waiver. We limited our analysis to DSH payments made to general, acute-care hospitals (defined in the American Hospital Association Annual Survey), which were assigned to their respective counties. We obtained county characteristics from the American Community Survey and County Health Rankings. All analyses were performed at the county level. The study followed the STROBE reporting guideline and was deemed not human participants research by institutional review boards at Weill Cornell Medical College and University of Pennsylvania Perelman School of Medicine.

We focused on 1 county-level measure that reflects care provided to low-income patients and is an explicit target of DSH programs (hospitals’ uncompensated care costs as a share of operating expenses), 1 county-level measure that is a major contributor to uncompensated care (percentage of uninsured residents), and 2 county-level measures of population health that reflect health-related disadvantage (age-adjusted premature mortality per 100 000 residents and age-adjusted percentage of adults reporting fair or poor health).

We plotted mean values of these measures among counties with the largest (top quartile) proportions of Black residents (based on self-report in the American Community Survey) relative to other counties (bottom 3 quartiles) across Medicare and Medicaid DSH payments per county resident. We then used ordinary least-squares regression to compare measures for disproportionately Black and other counties, holding Medicare and Medicaid DSH payments per resident constant.

Analyses were conducted using Stata/SE, version 16.1 (StataCorp LLC). All *P* values were from 2-sided tests, and results were deemed statistically significant at *P* < .05.

## Results

Counties with the largest proportions of Black residents (n = 588; mean, 28.6% Black residents in 2019) received a mean of $9 per resident in Medicare DSH payments and $52 per resident in Medicaid DSH payments, relative to other counties (n = 1766; mean, 2.5% Black residents in 2019), which received a mean of $4 per resident in Medicare DSH payments and $20 per resident in Medicaid DSH payments.

Disproportionately Black counties that received the same level of funding as other counties demonstrated higher rates of uncompensated care and health-related disadvantage (Figure). Holding Medicare DSH payments per resident constant, counties with the largest proportions of Black residents, relative to other counties, had significantly higher rates of uncompensated hospital care (difference, 2.0 [95% CI, 1.5-2.5] percentage points; *P* < .001), percentage of uninsured residents (difference, 2.5 [95% CI, 2.0-2.9] percentage points; *P* < .001), premature mortality (difference, 75.8 [95% CI, 65.0-86.6] deaths per 100 000 residents]; *P* < .001), and percentage of residents reporting poor or fair health (difference, 3.6 [95% CI, 3.1-4.1] percentage points; *P* < .001) (Table). Similar results were evident when Medicaid DSH payments were held constant; counties with the largest proportions of Black residents, relative to other counties, had significantly higher rates of uncompensated hospital care (difference, 1.4 [95% CI, 0.9-1.9] percentage points; *P* < .001), percentage of uninsured residents (difference, 2.3 [95% CI, 1.8-2.7] percentage points; *P* < .001), premature mortality (difference, 64.2 [95% CI, 54.6-73.8] deaths per 100 000 residents; *P* < .001), and percentage of residents reporting poor or fair health (difference, 3.6 [95% CI, 3.1-4.0] percentage points; *P* < .001).

**Figure.**
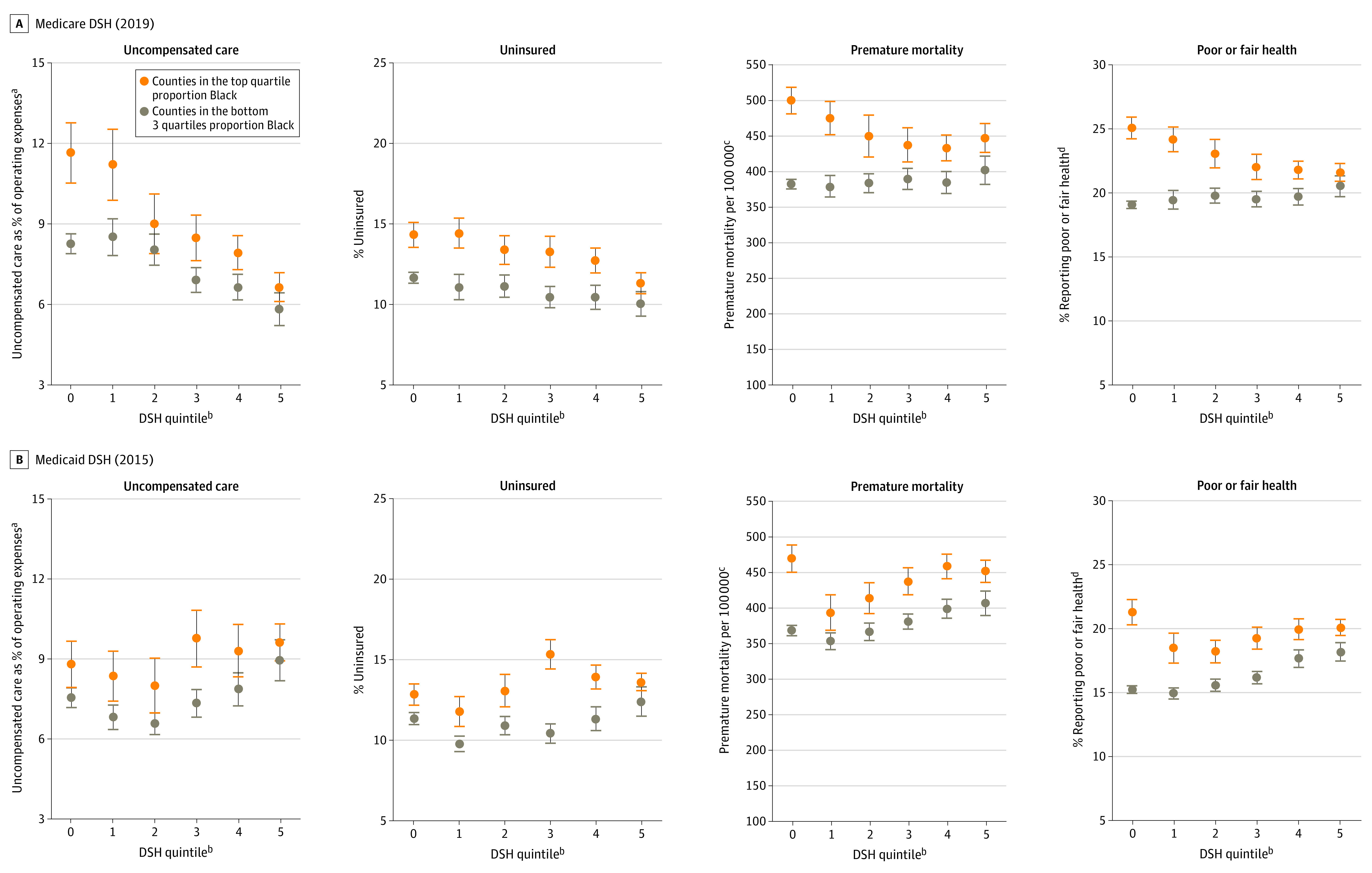
Association Between Disproportionate Share Hospital (DSH) Funding per Resident and County-Level Characteristics, by Race Error bars indicate 95% CIs. ^a^Reflects the mean value across hospitals in the county, weighted by each hospital’s number of beds. ^b^Reflects quintiles of DSH payments in dollars per county resident across counties with nonzero DSH allocations. Counties receiving zero dollars in DSH payments are shown in quintile 0. ^c^Number of deaths among residents younger than 75 years per 100 000 population (age adjusted). ^d^Percentage of adults reporting fair or poor health (age adjusted).

**Table.** Differences in County Characteristics by Race, Holding DSH Payments Constant

Characteristic	No.	Overall mean across counties (SD)	Difference in top quartile Black counties vs other counties, holding DSH funding constant (95% CI)^a^	*P* value
**Medicare DSH (2019)**				
Uncompensated care				
Uncompensated care share, %^b^	2354	8.1 (5.0)	2.0 (1.5-2.5)	<.001
Uninsured, %	2354	11.6 (5.0)	2.5 (2.0-2.9)	<.001
Population health				
Premature mortality per 100 000, No. deaths^c^	2352	403.7 (111.0)	75.8 (65.0-86.6)	<.001
Poor or fair health, %^d^	2354	20.3 (4.8)	3.6 (3.1-4.1)	<.001
**Medicaid DSH (2015)**				
Uncompensated care				
Uncompensated care share, %^b^	2377	7.9 (4.6)	1.4 (0.9-1.9)	<.001
Uninsured, %	2377	11.7 (4.9)	2.3 (1.8-2.7)	<.001
Population health				
Premature mortality per 100 000, No. deaths^c^	2372	391.9 (102.2)	64.2 (54.6-73.8)	<.001
Poor or fair health, %^d^	2377	16.9 (4.6)	3.6 (3.1-4.0)	<.001

Abbreviation: DSH, Disproportionate Share Hospital.

^a^ Reflects ordinary least-squares regression with categorical control for quintiles of DSH payments in dollars per county resident across counties with nonzero DSH allocations. Counties receiving zero dollars in DSH payments were placed in their own category.

^b^ Mean uncompensated care costs as shared of operating expenses across hospitals in county weighted by number of hospital beds.

^c^ Number of deaths among residents younger than 75 years per 100 000 population (age adjusted).

^d^ Percentage of adults reporting fair or poor health (age adjusted).

## Discussion

Among counties receiving the same level of DSH payments, disproportionately Black counties demonstrated higher levels of uncompensated care and worse population health. These findings suggest that DSH programs, by relying on measures of patient characteristics that reflect health care use, may structurally disadvantage communities that most require resources to improve population health. This study was limited in that it did not evaluate non-DSH supplemental payments and did not examine state-level heterogeneity in DSH allocations. Nonetheless, these findings suggest that policy makers should consider measures not based on health care use to ensure more equitable targeting of DSH payments or additional allocations to historically underserved communities.
